# A weak edge estimation based multi-task neural network for OCT segmentation

**DOI:** 10.1371/journal.pone.0316089

**Published:** 2025-01-03

**Authors:** Fan Yang, Pu Chen, Shiqi Lin, Tianming Zhan, Xunning Hong, Yunjie Chen

**Affiliations:** 1 School of Mathematics and Statistics, Nanjing University of Information Science and Technology, Nanjing, Jiangsu, China; 2 School of Computer Science, Nanjing Audit University, Nanjing, Jiangsu, China; 3 Center for Applied Mathematics of Jiangsu Province, Nanjing University of Information Science and Technology, Nanjing, Jiangsu, China; 4 The First Affiliated Hospital with Nanjing Medical University, Nanjing, Jiangsu, China; 5 Jiangsu International Joint Laboratory on System Modeling and Data Analysis, Nanjing University of Information Science and Technology, Nanjing, Jiangsu, China; Bayer Crop Science United States: Bayer CropScience LP, UNITED STATES OF AMERICA

## Abstract

Optical Coherence Tomography (OCT) offers high-resolution images of the eye’s fundus. This enables thorough analysis of retinal health by doctors, providing a solid basis for diagnosis and treatment. With the development of deep learning, deep learning-based methods are becoming more popular for fundus OCT image segmentation. Yet, these methods still encounter two primary challenges. Firstly, deep learning methods are sensitive to weak edges. Secondly, the high cost of annotating medical image data results in a lack of labeled data, leading to overfitting during model training. To tackle these challenges, we introduce the Multi-Task Attention Mechanism Network with Pruning (MTAMNP), consisting of a segmentation branch and a boundary regression branch. The boundary regression branch utilizes an adaptive weighted loss function derived from the Truncated Signed Distance Function(TSDF), improving the model’s capacity to preserve weak edge details. The Spatial Attention Based Dual-Branch Information Fusion Block links these branches, enabling mutual benefit. Furthermore, we present a structured pruning method grounded in channel attention to decrease parameter count, mitigate overfitting, and uphold segmentation accuracy. Our method surpasses other cutting-edge segmentation networks on two widely accessible datasets, achieving Dice scores of 84.09% and 93.84% on the HCMS and Duke datasets.

## 1 Introduction

Optical Coherence Tomography (OCT) technology can reconstruct the details of the retina and other ocular structures by obtaining high-resolution fundus images using near-infrared light and the principle of interference. The retina is primarily composed of various tissue layers, including the Nerve Fiber Layer (NFL), Inner Plexiform Layer (IPL), Inner Nuclear Layer (INL), Outer Plexiform Layer (OPL), Outer Nuclear Layer (ONL), Inner Segment (IS), Outer Segment (OS), and Retinal Pigment Epithelium (RPE). By examining the thickness of the various tissue layers, physicians can more easily assess the severity and progression of diseases such as diabetic macular edema [1], multiple sclerosis [2], and glaucoma [3]. Accurate segmentation of OCT fundus tissue is essential for calculating tissue thickness. In clinical practice, physicians often need to manually segment OCT images to determine tissue thickness. Due to common issues such as weak boundaries in OCT images, manual segmentation requires significant expertise and is time-consuming, with results that may lack reproducibility. Therefore, automated segmentation technology based on computer vision can provide physicians with stable and accurate segmentation results, offering quantitative information to aid in diagnosis.

Traditional segmentation methods have been extensively explored in the field of OCT retinal segmentation [4]. Ishikawa et al. [5] detected the peak or valley points on each A-scan using intensity gradients to locate the positions of different boundaries. They then applied curve fitting to these points to obtain continuous boundaries, which were subsequently used to generate the segmentation results. Lang et al. [6] extracted 27 features from the OCT data and input them into a random forest classifier, which generated boundary probabilities for each pixel. These probabilities were subsequently refined using a boundary refinement algorithm, leading to accurate segmentation results for exactly eight retinal layers. However, the aforementioned methods are overly reliant on carefully designed parameters, and they fail to meet clinical demands when dealing with large volumes of data [4].

With the continuous development of machine learning and image processing techniques, neural networks have emerged as prominent research directions in the domain of image classification, target detection. To solve the problems the issues mentioned above, many deep learning based segmentation methods have been proposed. Ronneberger et al. [7] proposed the U-Net network, which utilizes a structure that combines an encoder and a decoder and uses jump connections to improve segmentation performance. The encoder part resembles a common convolutional neural network, gradoublely reducing the spatial resolution of the input image and extracting high-level abstract features. The decoder part gradoublely restores the resolution and fuses the low-level features with the high-level features, thus improving the network accuracy.

**Fig 1:**
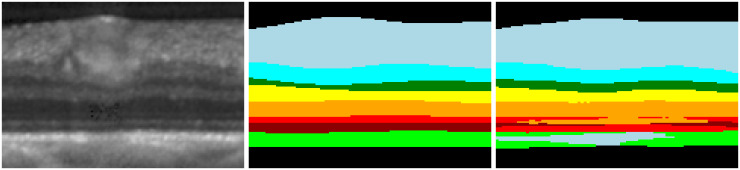
Predicted results of the TCCT [4] network. (A)the initial image (B)the ground truth (C)the segmentation results.

Several enhanced models, such as UNet++ [8] and TransUnet [9], have been developed based on the U-Net architecture for the analysis of medical images. However, deep learning networks encounter two challenges in this domain. Firstly, most models heavily rely on cross-entropy loss, which fails to preserve weak edge information [10], as shown in Fig 1. Secondly, due to the need for expert knowledge and patient privacy protection, the availability of labeled medical image data is limited, leading to models that are susceptible to overfitting [11].

This paper introduces a double-branch neural network using a shared encoder, where the outputs of the two branches are respectively used for image segmentation and boundary regression. The image segmentation branch uses the U-Net network structure, while the boundary regression branch uses a truncated signed distance function as the loss function, enabling the network to learn more boundary features and preserve weak edge details. We proposes a double-branch coupling module, which can reduce the semantic gap between the features learned by the two branches, allowing better fusion of information between the branches. However, due to the increase in the number of network branches, the parameter volume of the network increases, and with the scarcity of medical images, it is inadequate to support model training, which can lead to overfitting of the model. To solve this problem, this paper proposes a model pruning method based on channel attention. By using channel attention as a measure of convolutional kernel importance, the nonlinear relationship between convolutional kernels can be learned, unimportant convolutional kernels can be removed, and the redundancy of the model can be reduced, thereby avoiding the phenomenon of model overfitting. The main contributions of this paper are as follows:

- We propose a novel double-branch network that performs both image segmentation and boundary regression tasks separately. By incorporating this design, the network can effectively extract features from both the objects within the images and their boundaries, leading to improved performance in both tasks.
- To address the issue of noise caused by artificial labels applied to weak boundaries and preserve the topological structure of retinal layers, we employ a truncated signed distance function as the loss function for the boundary regression task. We also examined the characteristics of TSDF and designed an adaptive weight to enhance the network’s focus on boundary and misclassified regions.
- To capture non-linear interdependencies among channels and reduce the redundancy of convolutional kernels, we incorporate a channel attention layer after each convolutional layer. These layers acts as a measure of the importance of each filter, allowing for effective pruning of redundant filters. As a result, the parameter count of the network is reduced, reducing the risk of overfitting.

## 2 Related work

### 2.1 Single-task learning

With the rapid development of deep learning, convolutional neural networks (CNNs) have found widespread applications in diverse fields, including image classification [12], object detection [13], and image generation [14]. In particular, CNN-based medical image segmentation networks have made significant advancements [15].

Traditional CNN architectures, like Fully Convolutional Networks (FCNs) [16], often suffer from information loss during upsampling, impacting pixel-level segmentation accuracy [7]. To mitigate this, Ronneberger et al. [7] introduced the U-Net network, which employs an encoder-decoder structure with skip connections to fuse low-level and high-level features, although a semantic gap still exists [8]. Zhou et al. [8] later enhanced this with UNet++, a hierarchical model that captures more discriminative features at various levels. Additionally, Oktay et al. [17] proposed Attention U-Net, which incorporates attention mechanisms to focus on important regions of the image. Despite these advancements, U-Net struggles to leverage global contextual information, a limitation addressed by Chen et al. [9] with TransUnet, which integrates Transformer encoders for a more comprehensive understanding of semantic details. However, OCT fundus data presents a weak boundary issue, which the above methods fail to effectively address.

To better address the weak boundary issue in OCT fundus data, researchers have introduced a series of improved U-Net-based models. To explore the applicability of U-Net and its variants in OCT fundus image segmentation, Roy et al. [10] introduced the ReLayNet network, pioneering the use of deep learning for retinal layer segmentation. However, due to weak boundary issues in OCT fundus data, segmentation results from mainstream U-Net models and their variants often struggle to preserve the topology of retinal layers accurately. To address this issue, He et al. [18] proposed SR-Net, which comprises two cascaded deep networks. S-Net is designed to learn features from the original images for initial segmentation, while R-Net uses the segmentation labels learned by S-Net as inputs to further refine boundary positions and reconstruct the segmented image. This approach helps preserve the topological structure of the segmented image. Despite this, the learning task of R-Net is not direct boundary regression, leading to the model only implicitly learning boundary surfaces [19]. To improve the network’s ability to learn boundary features, many works have introduced boundary regression as an auxiliary task for the network to learn.

### 2.2 Multi-task learning

Single-task learning often fails to guide the network in learning boundary features effectively. To address this limitation, multi-task learning has become a primary approach in OCT fundus image segmentation, with the introduction of boundary regression playing a crucial role. This task enhances the model’s ability to accurately delineate retinal structures, thereby preserving fine details and maintaining topological integrity.

He et al. [19] use two projection heads on Res-U-Net to produce outputs for image segmentation and boundary regression, employing soft-argmax and smooth L1 loss for the latter. This multi-task learning improves segmentation performance by enhancing boundary detail capture; however, using the same codec for both tasks may hinder the model’s ability to fully represent both features, potentially reducing accuracy. Tan et al. [4] combine CNN and transformer techniques to leverage local and global information, proposing a boundary loss function with soft-argmax. While this enhances accuracy, a semantic gap remains between features from segmentation and boundary regression, and reliance on a single decoder restricts overall model potential. Our method uses two decoders to separately learn the tasks of image segmentation and boundary regression, thereby improving the model’s ability to extract these distinct features. Wang et al. [20] subtract deep features from shallow ones to extract edge information, introducing a Canny-based feature fusion module for boundary regression. Their method employs Kullback-Leibler scatter (KL scatter) to measure boundary column coordinates but treats neighboring coordinates as independent, ignoring horizontal dependencies. To address this, our method uses TSDF for boundary regression, which captures spatial relationships among neighboring pixels, thereby improving boundary delineation and overall segmentation performance.

Recent advancements in segmentation and boundary regression aim to help models retain detail and distinguish semantic features. However, a semantic gap between tasks persists, as most studies use a single encoder-decoder structure, limiting feature capture and hindering feature fusion [21]. Our method addresses this limitation by introducing dual decoders. Additionally, current boundary regression tasks often treat columns as independent units, overlooking their correlations, which can disrupt boundary topology. To better preserve topological features, we propose a TSDF-based boundary regression loss inspired by the level set method.

### 2.3 Network pruning

Complex networks frequently result in an upsurge in model parameters, leading to amplified training costs and vulnerability to overfitting. This issue is particularly pronounced in medical image data analysis, where the limited capacity of training sets makes models more susceptible to overfitting. To tackle these challenges, a number of scholars have employed pruning methods to enhance model efficiency and mitigate the risks associated with overfitting [22].

The prevailing pruning approach involves selecting and removing appropriate convolution kernels, with the crucial step being the selection of the convolution kernels to be pruned. Han et al. [23] proposed a method that utilizes L1 and L2 norms to evaluate the performance of convolution kernels, allowing for the identification and elimination of less relevant ones. The L1 and L2 paradigms offer a straightforward and intuitive method for quantifying the importance of convolutional kernels. However, these paradigms usually assess each kernel in isolation, overlooking potential interdependencies among them. In contrast, our approach leverages channel attention layers to capture the nonlinear relationships between different convolutional kernels, thereby reducing redundancy. Pruning convolutional kernels critically impacts feature extraction. While traditional methods prune and fine-tune model parameters post-training, they underperform if pre-training is inaccurate. Dinsdale et al. [11] address this limitation with Simultaneous Training and Model Pruning, selecting and pruning kernels during training in iterative cycles, allowing for precise control over the model’s architecture. We adopt this training approach in our method to achieve more effective control over the model’s architecture and enhance parameter optimization.

In recent years, many studies in the field of OCT fundus segmentation have opted for lightweight networks to extract features, aiming to avoid overfitting due to data scarcity and to meet clinical demands. For example, Tightly combined Cross-Convolution and Transformer(TCCT) [4] employs a CNN with a constant channel size of 32 and a lightweight transformer for feature extraction. RelayNet employs a CNN with a constant channel size of 64. However, this model simplification inevitably compromises the ability to extract features effectively. To effectively prune unimportant convolutional kernels while preserving model accuracy, we propose a channel attention-based model pruning strategy.

## 3 Proposed model

### 3.1 Overall

In order to enhance the accuracy of network segmentation, we propose a dual-branch architecture for image segmentation and boundary regression. In the boundary regression section, we introduce a signed distance to characterize boundary geometric features, which helps guide the network to focus on boundary feature information. This approach overcomes the limitations of weak boundaries and keep the topology of the retinal layers.

**Fig 2:**
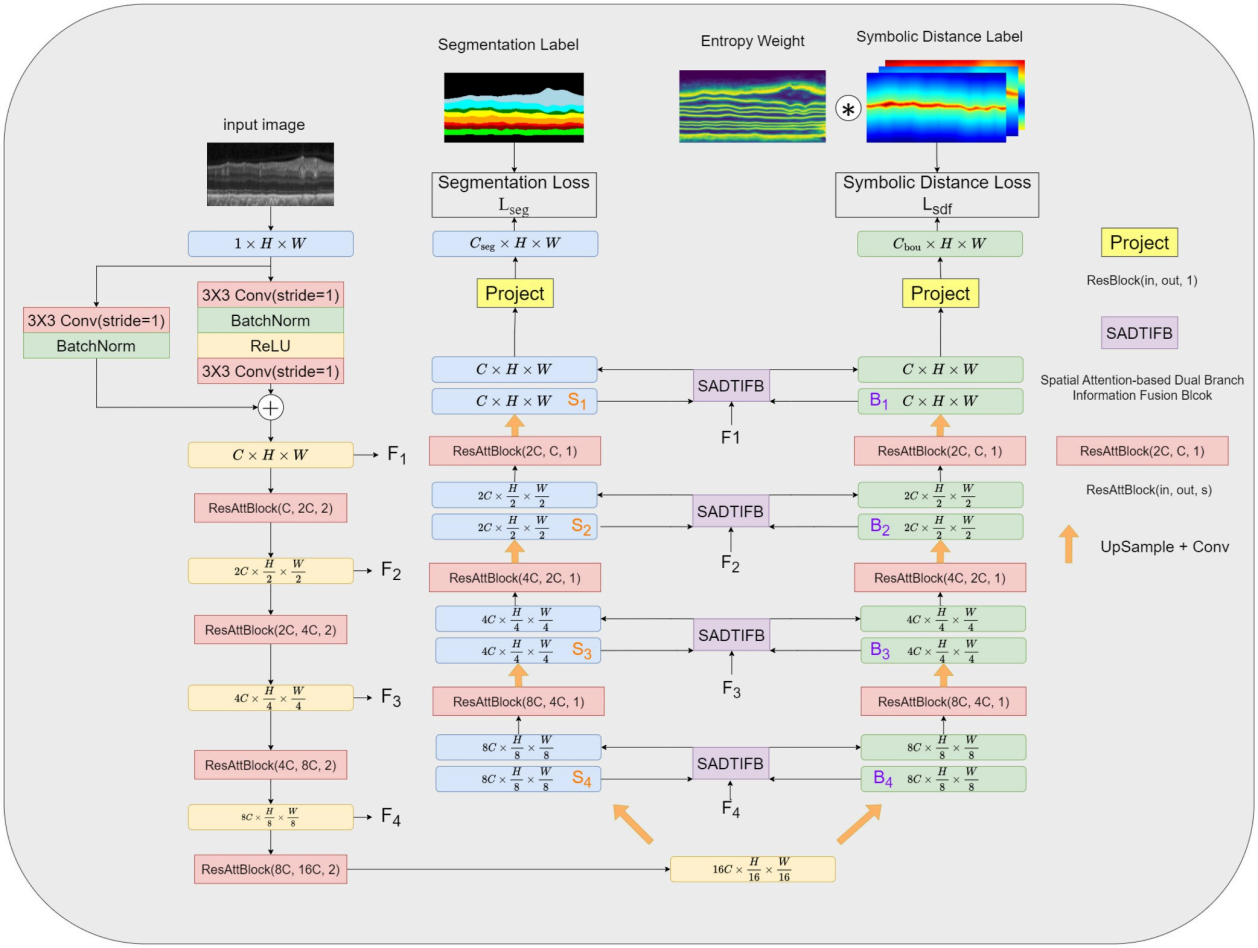
The structure of the proposed network. The proposed network is based on U-Net, consisting of a segmentation branch and a boundary regression branch. The segmentation branch extracts the overall feature information of the target, while the boundary regression branch focuses on extracting information related to the target’s boundaries.

**Fig 3:**
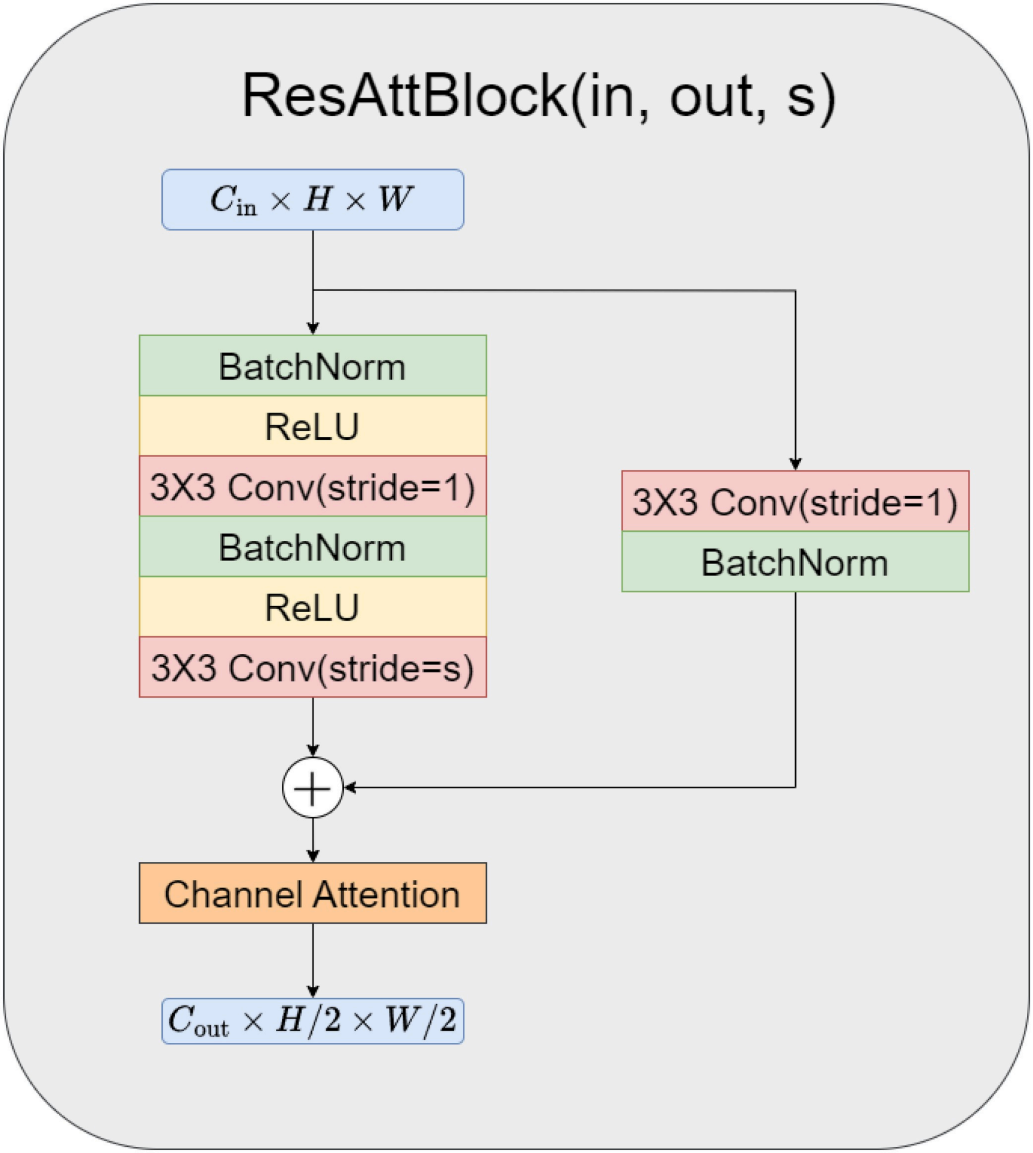
Schematic of the residual attention block.

To ensure consistency in the network features and reduce the number of model parameters, we utilize the same common encoding process for both tasks. However, for decoding, we use two different processes. The overall network structure, as shown in Fig 2, follows a similar design as the Res-UNet network, consisting of four layers for encoding and decoding. Each coding layer consists of a residual block and channel attention (ResAttBlock). The specific structure is shown in the Fig 3. These modules include convolutional layers, channel attention layers, batch normalization, and Rectified Linear Unit (ReLU) activation layers. The encoding layers are connected using max pooling for downsampling.

The high-level features obtained from the encoding process are fed into the two branches of the decoder in order to separate features. Both branches utilize a decoding structure similar to that of Res-UNet. Each branch of the network comprises four decoders, all of which are implemented as ResAttBlock. The outputs derived from each decoder in both branches are upsampled using bilinear interpolation, thus extracting features at different stages of the two paths, counted as segmentation features (*S*_*i*_) and boundary features (*B*_*i*_), respectively.

In order to effectively blend the feature information from both branches, we introduce a spatial attention based dual-branch information fusion block (SADBIFB), which addresses two important challenges at the same time. First, it helps reduce the semantic gap between encoding and decoding features. Second, it effectively fuses the decoding features from both the segmentation and regression branches, thus promoting a complementary relationship between the two branches. The SADBIFB couples the features obtained from the decoding layer (*F*_*i*_), the features obtained from the segmentation branch (*S*_*i*_), and the features obtained from the boundary regression branch (*B*_*i*_).

Ultimately, the outputs of the two branches are utilized for both image segmentation and boundary regression tasks. The output of the image segmentation branch indicates the probability of each pixel belonging to a specific category, with the pixel classified into the category with the highest probability. The boundary regression output indicates each pixel’s shortest distance to the category boundaries, which is known as TSDF [24]. To enhance the network’s focus on the boundary regions, we employ the Truncated Signed Distance Function (TSDF) [24] to guide the network in learning the boundary features.

The utilization of a double branch network may lead to an expansion in the number of model parameters and inadequate calibration data for medical image analysis, leading to overfitting issues in deep networks. In order to tackle these challenges, we propose a model pruning strategy that is based on channel attention. This strategy reduces the chances of overfitting and concurrently decreases the number of parameters in the model.

### 3.2 Spatial attention-based dual-branch information fusion block

**Fig 4:**
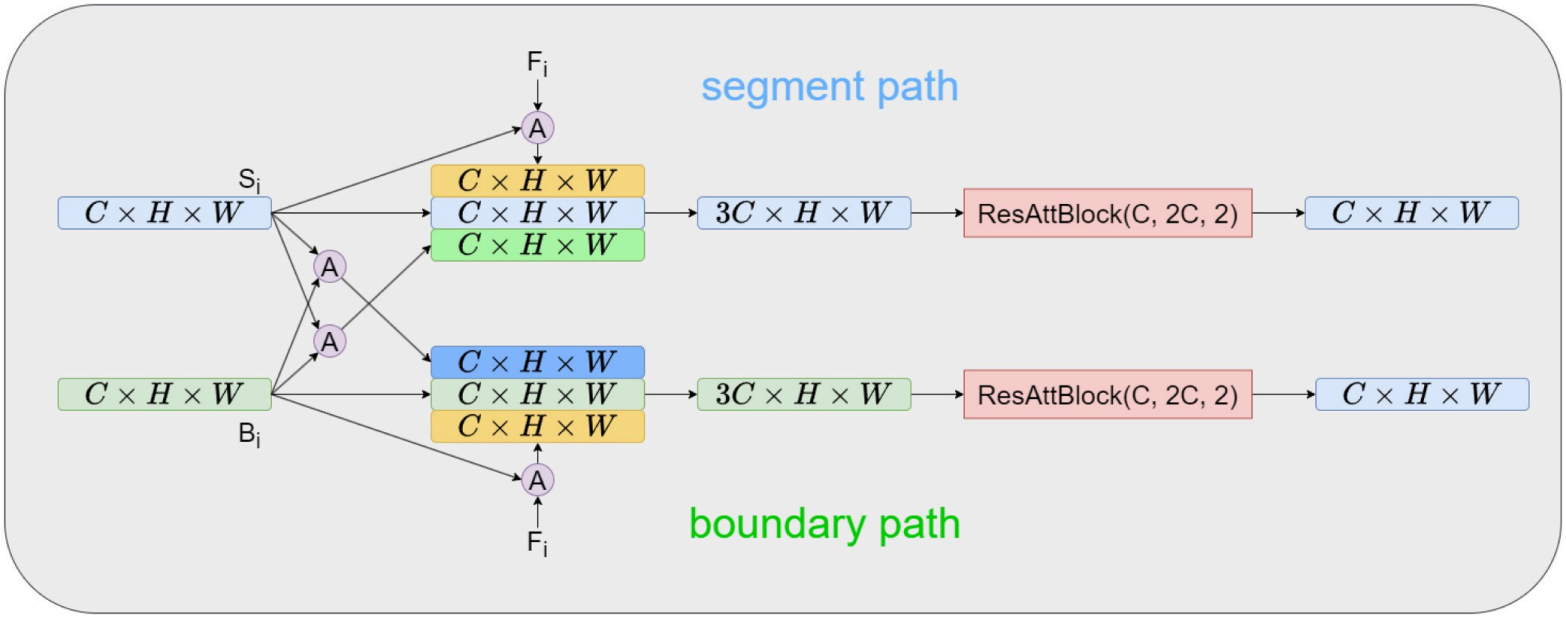
Schematic of SADBIFB.

In this section, we present SADBIFB as a solution to bridge the semantic gap between the two branches and effectively combine the feature information they provide. The structure of the SADBIFB is illustrated in Fig 4.

The inputs of SADBIFB consists of feature maps from the decoding layer (*F*_*i*_), the features obtained from the segmentation branch (*S*_*i*_), and the features obtained from the boundary regression branch (*B*_*i*_). These feature maps are of shape (*c* × *h* × *w*), where *c* represents the number of channels, *h* represents the image height, and *w* signifies the image width. To enhance the network’s spatial information ability, this study incorporates a spatial attention gate [17], which selectively focuses on informative spatial regions during the information fusion process.

**Fig 5:**
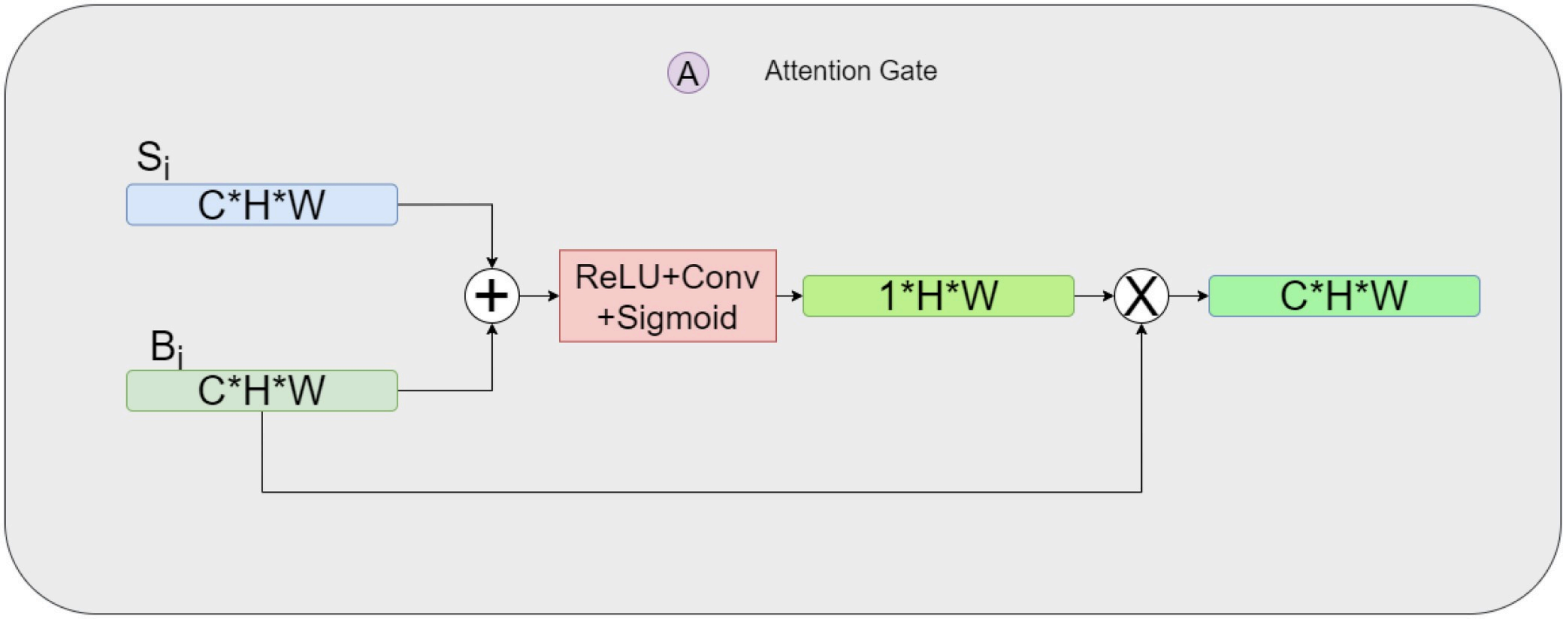
Schematic of the spatial attention gate [17]. Taking the segment branch as an example, this gate extracts pertinent information from the boundary regression branch *B*_*i*_ which is crucial for segmentation branch.

In the segmentation branch, we utilize two spatial attention gates to extract effective encoded features and boundary regression features. The same approach is taken in the boundary regression branch as well. For instance, in the segmentation branch, the boundary regression feature space attention gate (shown as the red area in Fig 4 is used. This gate applies spatial attention to the features in *S*_*I*_ and *B* to extract the spatial importance of feature information. This spatial importance is then applied to *B*_*I*_ to extract important boundary regression features in the segmentation branch, which provides more effective boundary regression feature information. The specific structure of this process is depicted in Fig 5. The attention gate function *G*(*S*_*i*_, *B*_*i*_) of the example is defined as follows:

$$\begin{array}{c} G\left(S_{i},B_{i}\right)=\sigma \left(w_{3}\delta \left(w_{1}S_{i}+w_{2}B_{i}\right)\right)\cdot B_{i} \end{array}$$ (1)

Here, $\left\{w_{1},w_{2}\right\}\in R^{\frac{c}{2}\times c}$ and $w_{3}\in R^{1\times \frac{c}{2}}$ are the parameters that need to be learned. Here, *c* represents the number of channels in the feature maps.

The function takes two inputs, *S*_*i*_ and *B*_*i*_, and computes the attention gate output. The attention gate function involves two activation functions: *δ* and *σ*. The activation function *δ* uses ReLU, which ensures that only positive values are passed through. The activation function *σ* uses the sigmoid function, which squashes input values between 0 and 1. The element-wise multiplication operator (⋅) is used to perform a pointwise multiplication between the weight vector and the feature map along the channel dimension. This helps in weighting the importance of boundary regression features and improve the ability of the segmentation branch. The other three spatial attention gates follow the same structure as this example.

Using the segmentation branch as an example, we employ a spatial attention mechanism to extract features from encoding features and boundary regression features. These features are then concatenated with segmentation features in the channel dimension. The fused features are further processed through convolutional layers.

### 3.3 Truncated signed distance function for boundary regression

In the given method, we are calculating the distance between each pixel and the nearest boundary pixel for each category Ω_*k*_. In order to make the network more focused on the features of the boundary, we use the truncated signed distance function(TSDF) as the objective function for boundary regression branch learning. The TSDF is defined as follows:

$$\begin{array}{c} \text{d}\left(x,\partial \Omega_{k}\right)=\{\begin{array}{cc} -\text{min}\left(d\left(x,\partial \Omega_{k}\right),\tau \right), & \text{if}\;x\in \Omega_{k}\\ \text{min}\left(d\left(x,\partial \Omega_{k}\right),\tau \right), & \text{if}\;x\in \Omega_{k}^{c} \end{array} \end{array}$$ (2)

Here, *x* represents a pixel in the image, *τ* represents truncation distance,and ∂Ω_*k*_ is the set of pixels that form the boundary of the *k*th target. The function *d*(*x*, *ω*) calculates the Euclidean distance between the pixel *x* and the pixel *ω*. The TSDF assigns negative values to pixels inside the target Ω_*k*_ and positive values to pixels outside Ω_*k*_. The negative value represents the distance to the boundary pixel inside the target, whereas the positive value represents the distance to the boundary pixel outside the target. Next, for training stability, we normalize the distance *d*(*x*, ∂Ω_*k*_) using the following formula:

$$\begin{array}{c} \hat{d}\left(x,\partial \Omega_{k}\right)=\frac{d(x,\partial \Omega_{k})}{\underset{x\in \Omega_{k}\cup \Omega_{k}^{c}}{\text{max}}\;|d(x,\partial \Omega_{k})|} \end{array}$$ (3)

Here, $\hat{d}\left(x,\partial \Omega_{k}\right)$ represents the normalized distance, which we refer to as *d*^*k*^ for convenience. This normalization scales the truncated distance values between the range of [-1, 1]. We observed that the difference between the maximum and the second-largest values of the TSDF, computed by the network for pixel points near the boundary and in misclassified regions, is small. The former is due to the inherent characteristics of the true TSDF corresponding to the pixel points themselves, while the latter indicates that the network has not correctly learned the topology of the image. To encourage the network to focus more on boundaries and misclassified regions, we propose an adaptive weighting scheme based on information entropy. This scheme leverages the maximum and second-largest values of the TSDF, as described by the following formula:

$$\begin{array}{c} d_{\text{max}\;1}\left(x\right)=\underset{k\in 1,2,\ldots ,K}{\text{max}}\;d^{k}\left(x\right) \end{array}$$ (4)

$$\begin{array}{c} d_{\text{max}\;2}\left(x\right)=\underset{k\in 1,2,\ldots ,K\backslash k_{\text{max}\;1}}{\text{max}}\;d^{k}\left(x\right) \end{array}$$ (5)

where *k*_max 1_ is the index that maximizes *d*^*k*^(*x*):

$$\begin{array}{c} k_{\text{max}\;1}=\text{arg}\;\underset{k\in 1,2,\ldots ,K}{\text{max}}\;d^{k}\left(x\right) \end{array}$$ (6)

For each pixel point *d*_max 2_(*x*), *d*_max 2_(*x*) the first two maxima are converted to probability values using the softmax function:

$$\begin{array}{c} p_{1}\left(x\right)=\frac{\text{exp}(d_{\text{max}\;1}\left(x\right))}{\text{exp}\left(d_{\text{max}\;1}\left(x\right)\right)+\text{exp}\left(d_{\text{max}\;2}\left(x\right)\right)} \end{array}$$ (7)

$$\begin{array}{c} p_{2}\left(x\right)=\frac{\text{exp}(d_{\text{max}\;2}\left(x\right))}{\text{exp}\left(d_{\text{max}\;1}\left(x\right)\right)+\text{exp}\left(d_{\text{max}\;2}\left(x\right)\right)} \end{array}$$ (8)

Based on these probability values, the information entropy *w*(*x*) is calculated:

$$\begin{array}{c} w\left(x\right)=\frac{-(p_{1}\left(x\right)\;\text{log}\;p_{1}\left(x\right)+p_{2}\left(x\right)\;\text{log}\;p_{2}\left(x\right))}{\text{log}\;2} \end{array}$$ (9)

**Fig 6:**
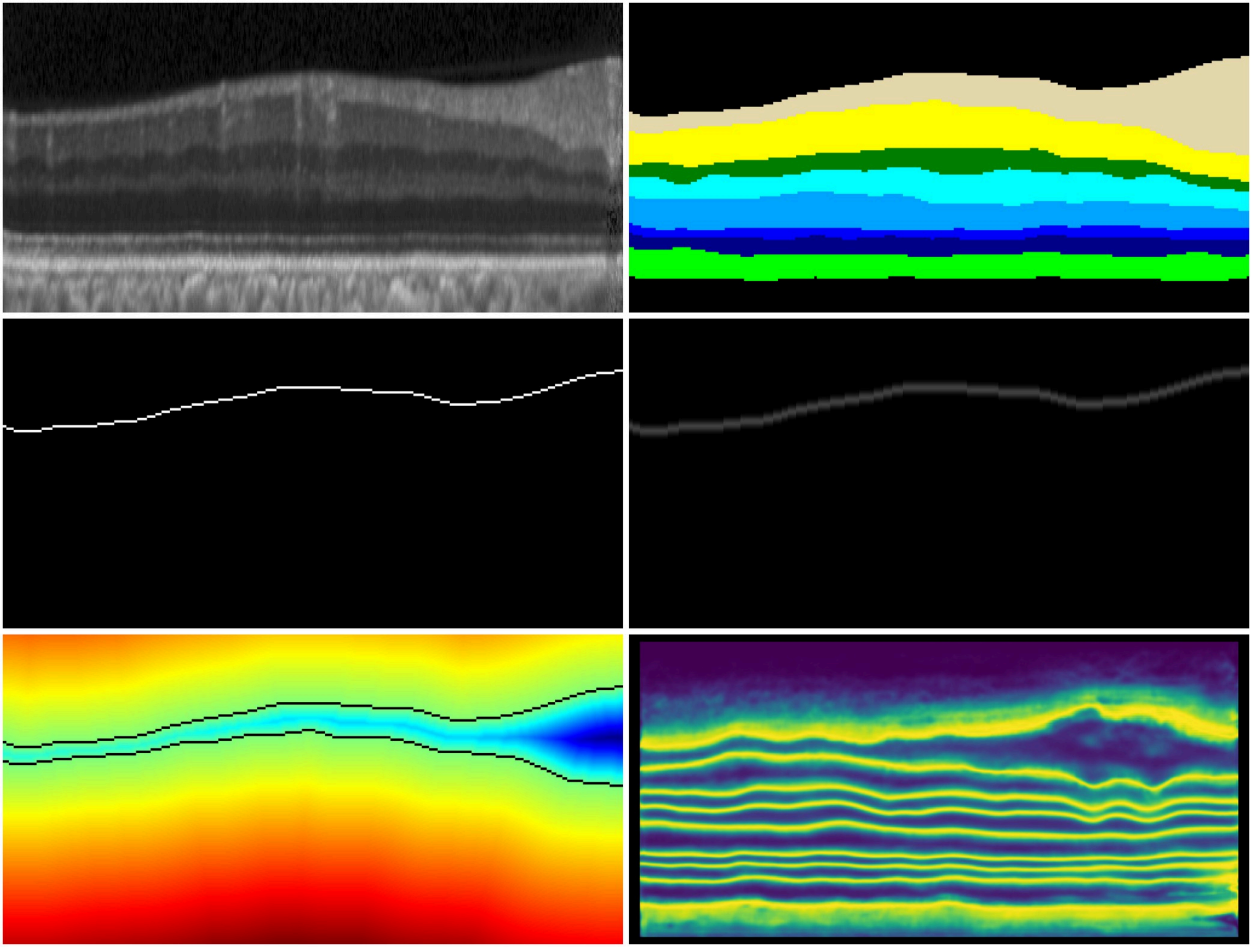
Visualized results of different boundary labels. The first row presents the OCT fundus image and its corresponding pixel-level label. The second row shows the boundary label and the label smoothed with a Gaussian kernel. The third row features the TSDF-based label (The boundary (zero TSDF) is represented as black) and the information entropy weights.

Fig 6 illustrates the boundary labels employed in [4, 19], which are presented in the second row. The third row displays the SDF labels that we utilize, along with the adaptive weights applied. Due to the unique topology of the retinal layer, He et al. [19] employed one-hot encoding for boundary labels and regressed the boundary coordinates of each column. They utilized KL divergence loss to enable the network to learn multiple independent distributions of the column coordinates. In contrast, Tan et al. [4] highlighted that there is inherent uncertainty in the labels. They employed soft-argmax to enable the model to learn a smooth distribution with higher probability near the boundaries. However, they noted that the boundary coordinate distributions of different columns are not independent, and there is a significant degree of dependence between adjacent columns. SDF describes the distance from each point in the image to the target boundary. Distances inside the target are positive, while those outside are negative, with the zero level set representing the target boundary (marked in black in the figure). This approach makes it easier for the network to capture boundary features. Additionally, SDF better preserves slender topologies, leading to improved segmentation in thinner retinal layers. It is worth noting that our method does not generate spurious boundaries in complex boundary areas.

The loss function in our model is composed of the segmentation loss (*L*_*seg*_) and the boundary regression loss (*L*_*bou*_), and it is calculated as follows:

$$\begin{array}{c} L=L_{seg}+\lambda L_{bou} \end{array}$$ (10)

Here, *L*_*seg*_ represents the segmentation loss, which is computed using the cross-entropy loss and Dice loss for K classes. The equation for *L*_*seg*_ is given as:

$$\begin{array}{c} L_{seg}=L_{seg}+L_{dice} \end{array}$$ (11)

$$\begin{array}{c} L_{ce}=\frac{1}{N}\sum_{i=1}^{N}\sum_{k=1}^{K}\frac{1}{HW}\sum_{j=1}^{HW}w\left(x_{j}\right)y_{i}^{k}\left(x_{j}\right)\;\text{log}\;p_{i}^{k}\left(x_{j}\right) \end{array}$$ (12)

$$\begin{array}{c} L_{dice}=\sum_{k=1}^{K}\sum_{i=1}^{N}(1-\frac{\sum_{j=1}^{HW}2y_{i}^{k}\left(x_{j}\right)p_{i}^{k}\left(x_{j}\right)}{\sum_{j=1}^{HW}y_{i}^{k}\left(x_{j}\right)+\sum_{j=1}^{HW}p_{i}^{k}\left(x_{j}\right)}) \end{array}$$ (13)

In the equation above, *N* represents the number of pixels in the image, $y_{i}^{k}$ is the ground truth, and $p_{i}^{k}$ is the output of the segmentation branch. The boundary regression loss *L*_*bou*_ is computed as the mean square error (MSE) between the output of the boundary regression branch and the truncated signed distance function. The equation for *L*_*bou*_ is given as:

$$\begin{array}{c} L_{bou}=\frac{1}{N}\sum_{i=1}^{N}\sum_{k=1}^{K}\frac{1}{HW}\sum_{j=1}^{HW}w\left(x_{j}\right){\left(s_{i}^{k}\left(x_{j}\right)-d_{i}^{k}\left(x_{j}\right)\right)}^{2} \end{array}$$ (14)

Here, $s_{i}^{k}$ is the output of the boundary regression branch, and $d_{i}^{k}$ represents the truncated signed distance function of the original label.

### 3.4 Model pruning with channel attention

The number of parameters in our proposed two-branch network has increased by approximately 5.68 million parameter compared to the original U-Net network. Furthermore, limited medical image training set can lead to overfitting. To address this issue, we propose a channel attention-based pruning scheme. According to the lottery ticket hypothesis [22], a dense and large network contains a smaller subnetwork that can achieve equal or higher accuracy through training. By removing redundant parameters, we can significantly reduce the total parameter count in the model without compromising its accuracy. This approach helps mitigate the problem of overfitting.

Deep networks frequently employ a large number of convolutional kernels in their network architecture to improve their capacity for capturing image features. However, this strategy often results in parameter redundancy. In order to tackle the limitation of previous research, Behrad et al. [25] proposed a method to reduce parameter redundancy by utilizing the L1 and L2 norms to determine the importance of each feature channel. By removing the convolutional kernels corresponding to low importance channels, the researchers successfully reduced the number of model parameters. However, this approach only considers the importance of convolutional kernels based on their corresponding channels, neglecting the nonlinear relationship between these kernels. As a result, it fails to effectively eliminate redundant information between channels [26]. To address this limitation, we introduce the channel attention mechanism in this study. By incorporating the channel attention mechanism, the model can autonomously learn and select the most valuable feature channels. This allows for the capture of nonlinear relationships between these channels, specifically the nonlinear connections between convolutional kernels.

**Fig 7:**
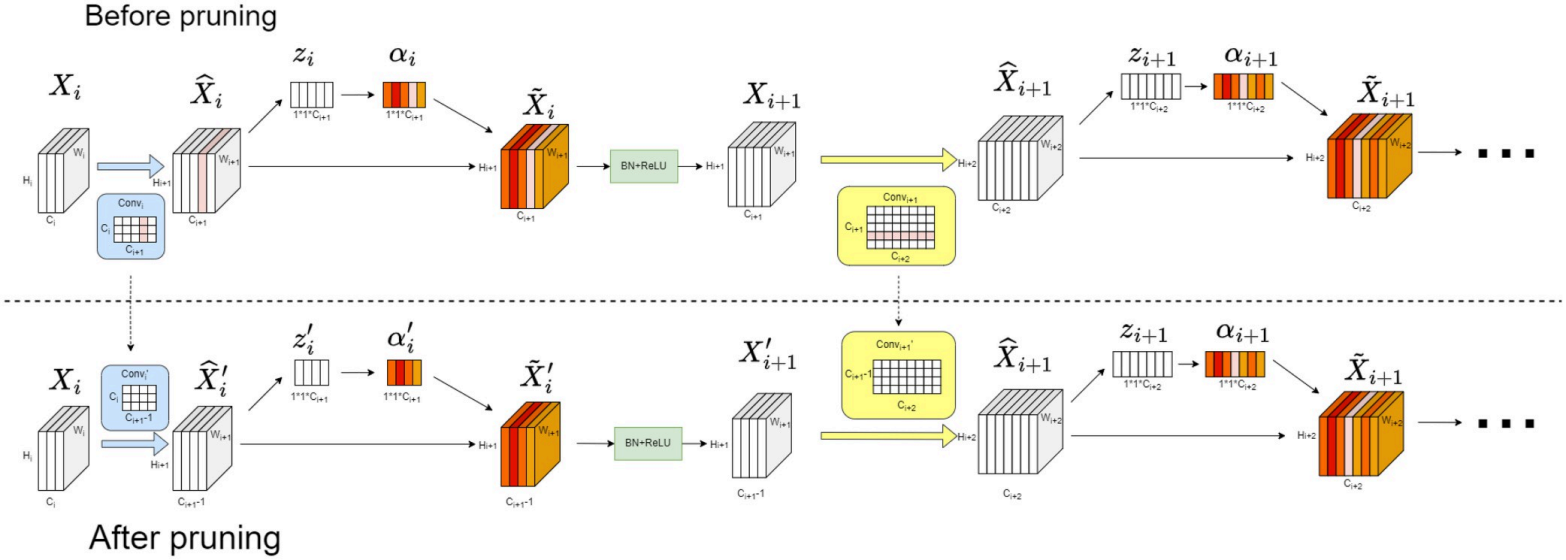
Schematic of the model pruning. The pink channels in the figure represent the channels that need to be pruned.

As depicted in Fig 7, the CA model adds a layer of channel attention after each convolutional layer. Let $X_{i}\in R^{c_{i}\times h_{i}\times w_{i}}$ denote the feature map of the input of the *i*th CA model followed by the *i* + 1th CA model in each branch. $Conv_{i}\in R^{c_{i+1}\times c_{i}\times k_{i}\times k_{i}}$ is the convolutional kernel, and $\hat{x_{i}}\in R^{c_{i+1}\times h_{i+1}\times w_{i+1}}$ is the corresponding output. To mitigate the influence of spatial information on channel attention, global max pooling and global average pooling are initially applied to compress the feature map from *C* × *W* × *H* to *C* × 1 × 1, as illustrated in the following formula:

$$\begin{array}{c} {z_{i}}_{c}=\underset{m,n}{\text{max}}\;X_{c}\left(m,n\right)+\frac{\sum_{m=1}^{h}\sum_{n=1}^{w}X_{c}\left(m,n\right)}{h\times w} \end{array}$$ (15)

Here, *z*_*i*__*c*_ represents the information in the *c*th channel of the feature map *z*_*i*_, and *X*_*c*_(*m*, *n*) represents the value at position (*m*, *n*) in the *c*th channel of the feature map. Then, non-linear interactions between channels are learned through the next two fully connected layers, as shown in the following formula:

$$\begin{array}{c} \alpha_{i}=\sigma \left(w_{2}\delta \left(w_{1}z_{i}\right)\right) \end{array}$$ (16)

In this formula, $w_{1}\in R^{\frac{c_{i+1}}{r}\times c_{i+1}}$, $w_{2}\in R^{c_{i+1}\times \frac{c_{i+1}}{r}}$ and *r* represents the compression ratio (typically set to 4). $\alpha_{i}\in R^{c_{i+1}\times 1\times 1}$ represents the weights assigned to each channel. Finally, the weights *α*_*i*_ and features $\hat{X_{i}}$ are transformed into points based on their spatial dimensions to acquire $\tilde{X_{i}}$.

Before evaluating the importance of convolutional kernels, it is necessary to pre-train the model for 100 epochs to allow the model to initially learn from the data. The importance of each convolutional kernel is determined based on the attention *α*_*i*_ of its corresponding channel. As the channel attention *α*_*i*_ is data-driven, it is not fixed after training the model; rather, it varies with changes in the input data. To overcome this, the dataset is divided into batches of equal size. Each batch is then fed into the network to obtain the *α*_*i*_ values for each convolutional layer in that batch. The final importance score for each convolutional kernel is calculated by summing up the *α*_*i*_ values obtained from all the batches. To obtain a subnetwork, the convolutional kernel importance metrics are sorted in ascending order, and the top *r*% kernels are selected and removed. Zhu et al. [27] found that the features of the shallow channels are more diverse than those of deep channels, based on the feature similarity matrix. To address this, we group and rank the convolutional kernels based on their depth, then prune the bottom *r*% of the least important kernels in each group. After fine-tuning, we prune the remaining *r*% of the kernels, repeating this process five times. The model with the best performance is then selected for retention.

Fig 7 illustrates the process of pruning a convolutional kernel. Once identified, we proceed to crop the specific convolutional kernel along with its associated feature channels. Additionally, we adjust the number of channels for each convolutional kernel in the next CA model to maintain the consistency of the network architecture.

However, it is important to note that the subnetwork obtained from the previous step is highly sensitive to initialization. To preserve the image features learned by the original network as effectively as possible, the training parameters of the original network are assigned to the unpruned parameters in the subnetwork as initialization. This ensures that the learned knowledge is retained. The subnetwork is then fine-tuned for an additional 20 epochs to further optimize its performance, resulting in the final model.

## 4 Experiments setup

### 4.1 Datasets

The data utilized in this study is sourced from two datasets, namely HCMS(https://iacl.ece.jhu.edu/index.php?title=Resources) and DUKE(https://people.duke.edu/~sf59/Chiu_BOE_2014_dataset.htm) dataset.

The HCMS dataset provides fundus OCT images of 21 multiple sclerosis patients and 14 normal individuals, with each dataset containing 49 B-scans. In total, the dataset comprises 1,715 images. The size of each image element is 496 × 1024. In order to reduce the computational effort, the images were subjected to a flatting operation using the same method as in [6], and then we cropped the redundant Choroid and Vitreous Body and resize to 256 × 512. We selected the last six HC volumes and the last nine MS volumes for training the model, while the remaining 20 volumes were reserved for testing.

The DUKE dataset includes 10 diabetic macular edema patients, each with 11 B-scans, totaling 100 images. Each image has a pixel size of 496 × 768. We used the first five patients for training and the last five patients for testing. Notably, we did not apply a flattening operation to this dataset. Instead, we directly cropped the excess choroid and vitreous and resized the images to 256 × 512.

The HCMS dataset includes retinal images from different patients, containing both healthy retinal images and images with pathological regions. This diversity allows the model to be exposed to a broader range of image types during training, enabling it to learn how to segment various retinal regions. On the other hand, the DUKE dataset specifically focuses on diabetic macular edema (DME) lesions, which is crucial for training the model’s ability to segment specific pathological areas. Conducting experiments on these two datasets ensures that the model can maintain high segmentation accuracy when faced with different types of images and various pathological conditions, thereby validating its generalization ability.

### 4.2 Training and evaluation metrics

To quantitatively evaluate our model and compare it with U-Net [7], Attention U-Net [17], UNet++ [8], Res-UNet [28], AttUnet [17], TransUnet [9], FCRN [19] and RelayNet [10], we employed the Dice, IOU as evaluation criteria. These two evaluation criteria is commonly utilized to quantify the similarity between model predictions and corresponding labels. It is computed by taking the intersection of the predicted and labeled regions and dividing it by the union of these two regions. We calculated the dice scores for each category and calculated the average dice score and iou score for all categories.

### 4.3 Training details

We perform five-fold cross-validation on the training set, using 80% of the data for training and 20% for validation in each fold. The model selected for the final evaluation is the one that achieves the highest mean Dice score (mdice) on the validation set. To manage computational costs, we resize both datasets to 256 × 512 pixels. During training, we apply random cropping to 256 × 256 pixels. For validation and testing, we retain the 256 × 512 pixel size. Our data augmentation strategy includes random horizontal and vertical flips, as well as random brightness and contrast adjustments, each with a probability of 0.5. The Adam optimizer was employed as the model’s optimizer, and the Cosine Annealing Learning Rate technique was utilized to adjust the learning rate. The learning rate started at 5e-4 and was reduced to 5e-6. The weight decay is set to 1e-5. During the training process, the batch size was set to 8, while during the testing process, it was set to 1. All experiments were trained for 100 epochs.

## 5 Experiments results and discussion

### 5.1 Segmentation results

To demonstrate the superiority of our model, we compared it with other popular image segmentation models. These models can be divided into three groups: traditional model, single task models and double task models. The traditional model include AUtomated Retinal Analysis(AURA) [6]. The single task models include U-Net [7], Attention U-Net [17], UNet++ [8], Res-UNet [28], and TransUnet [9]. The multi-task networks specifically designed for retinal layer segmentation include ReLayNet [10] and FCRN [19].

The segmentation results are shown in Table 1 for the HCMS dataset and in Table 2 for the Duke dataset. We set the number of channels for the single-task network to [32, 64, 128, 258, 512], following the conventional setting. For multitasking, we adhere to the original paper’s settings, which utilize a lightweight network with all channel numbers set to 64. Our network is configured to [32, 64, 128, 128, 128]. To compare the effect of parameter count on model accuracy, we also set the number of channels for the FCRN network to [32, 64, 128, 258, 512] for training.

**Table 1:** Dice scores for each tissue type in the HCMS dataset, as well as the average Dice score and average IoU for each tissue type.

Model	Params	NFL	IPL	INL	OPL	ONL	IS	OS	RPE	Dice	IoU
AURA [6]	/	90.78%*	92.60%*	83.96%*	83.39%*	93.19%*	82.82%*	85.05%*	87.39%*	87.40%*	78.70%*
U-Net [7]	7.85	92.61%*	94.19%*	86.95%*	89.25%*	94.32%*	85.79%*	86.76%*	91.51%*	90.17%*	82.49%*
Attention UNet [17]	8.72	92.01%*	93.93%*	87.13%*	89.39%*	94.56%*	86.43%*	87.01%*	91.27%*	90.22%*	82.52%*
UNet++ [8]	9.16	93.23%*	94.79%*	87.68%*	89.93%*	94.49%*	86.31%*	86.10%*	91.50%*	90.50%*	83.11%*
Res-UNet [28]	13.16	93.28%*	94.92%	88.07%*	90.30%*	94.96%*	86.93%*	86.95%*	91.45%*	90.86%*	83.60%*
Trans-UNet [9]	125.9	92.75%*	94.22%*	87.84%*	90.10%*	94.78%*	86.79%*	87.69%	91.13%*	90.71%*	83.34%*
ReLayNet(tiny) [10]	1.26	92.91%*	94.64%*	87.55%*	90.05%*	94.79%*	86.27%*	86.83%*	91.59%*	90.58%*	83.20%*
FCRN(tiny) [19]	1.38	93.37%*	94.91%	88.05%*	90.42%	95.02%	86.87%*	86.87%*	91.88%*	90.92%*	83.27%*
FCRN [19]	13.19	93.26%*	94.80%*	88.07%	90.36%*	95.02%	86.78%*	87.04%*	92.04%*	90.92%*	83.72%*
MTAMN(Ours)	6.64	93.44%	**94.95%**	**88.24%**	90.40%	94.94%	87.09%	87.18%	91.67%	90.99%	83.81%
MTAMNP(Ours)	2.07	**93.45%**	94.92%	88.16%	**90.43%**	**95.10%**	**87.22%**	**87.64%**	**92.27%**	**91.15%**	**84.06%**

* refers to the p-value of the paired t-test between each method and the proposed model(*: P<0.05).

**Table 2:** Dice scores for each tissue type in the DUKE dataset, as well as the average Dice score and average IoU for each tissue type.

Model	Params	RNFL	GCIPL	INL	OPL	ONL	IS	OS-RPE	Dice
U-Net [7]	7.85	87.56%*	90.31%*	77.26%*	77.60%*	93.80%*	90.02%*	83.70%*	85.75%*
Att-UNet [17]	8.72	87.30%*	91.13%*	80.67%*	78.14%*	94.14%*	89.44%*	85.56%*	86.63%*
UNet++ [8]	9.16	88.24%*	91.52%*	80.27%*	79.34%*	94.33%*	90.26%*	87.21%*	87.31%*
Res-UNet [28]	31.55	85.94%*	89.86%*	80.20%*	78.33%*	94.12%*	89.47%*	86.52%*	86.35%*
FCRN(tiny) [19]	1.38	88.13%*	90.93%*	80.63%*	79.36%*	94.69%*	89.72%*	86.57%*	87.15%*
MTAMNP(Ours)	2.07	**88.69%**	**92.29%**	**83.65%**	**81.20%**	**95.09%**	**90.34%**	**87.38%**	**88.38%**

* refers to the p-value of the paired t-test between each method and the proposed model(*: P<0.05).

The U-Net network [17] is currently the dominant method in the field of medical segmentation, and most of the current segmentation networks are based on variations of U-Net networks. To address the issue of U-Net overfitting, the Res-UNet network [28] incorporates the Res module to capture feature residouble information. UNet++ [8] utilizes multi-layer networks to bridge the semantic gaps between codecs, which cannot be overcome by the original U-Net skip connections, resulting in improved accuracy. By employing skip connections based on a spatial attention mechanism, Attention U-Net [17] reduces semantic redundancy between encoding and decoding, further enhancing segmentation accuracy. TransUnet leverages the Transform mechanism to extract the final result of the encoding layer and incorporate spatial information, leading to improved segmentation accuracy. However, this method only extracts features from the final encoding result and introduces additional parameters.

To enhance the network’s ability to extract boundary features, RelayNet [10] is designed with an adaptive weighting mechanism that takes into account both the image gradient magnitude and the number of categories. This approach enhances the model’s sensitivity to boundaries and increases the emphasis on foreground regions, leading to more accurate segmentation results. FCRN [19] employs a double head project to obtain boundary feature information while performing segmentation, but single branch limits the accuracy improvement.

Our proposed method utilizes a coupling mechanism that integrates segmentation and boundary regression, enabling us to restore boundary information while performing segmentation. The synergy between the two components results in optimal outcomes.

From Table 1, we can observe that mainstream segmentation networks have achieved notable results. Among them, the Res-UNet network stands out by expanding the receptive field through residual connections with convolutions using different dilation rates. This approach effectively mitigates the gradient vanishing and explosion issues caused by having too many channels, thereby improving model accuracy, making Res-UNet the most effective among mainstream segmentation networks.

However, it is noteworthy that the number of parameters in TransUnet far exceeds that of other networks, yet its IoU only reaches 83.34%, making it less favorable for clinical use. In networks specifically designed for fundus segmentation, we set the number of channels to 64 following the original paper’s settings. Consequently, the parameter counts for RelayNet (tiny) and FCRN (tiny) were 1.26M and 1.38M, respectively. However, the limited number of parameters restricted the model’s capability.

To verify our hypothesis, we modified the number of channels in FCRN to [32, 64, 128, 256, 512], resulting in a total of 13.9M parameters. Experimental results showed that the IoU improved by 0.45% compared to the tiny version of FCRN. Our method achieved the best results across all evaluated datasets, with the number of parameters being only 2.07M.

The Duke dataset, characterized by edema and severe deformation, coupled with its smaller size and the presence of some missing annotations, led to significantly lower accuracy for all models compared to the HCMS dataset. It is also important to note that we did not flatten the DUKE dataset, which resulted in a large amount of background area in the images, which may be one of the reasons for the mediocre results of both the methods we compared and our own. However, all experiments were performed in a uniform setup. From the Table 2, it can be seen that UNet++ demonstrates higher accuracy for this complex deformation data, primarily due to its ability to fuse multi-scale features. Additionally, the boundary regression task of the FCRN network somewhat enhances the accuracy of segmentation tasks on slender topologies. Our network further improves learning capability on complex deformation data through its dual-branch architecture and weighted TSDF approach. Our method achieved a 3.57% improvement in IoU compared to U-Net.

**Fig 8:**
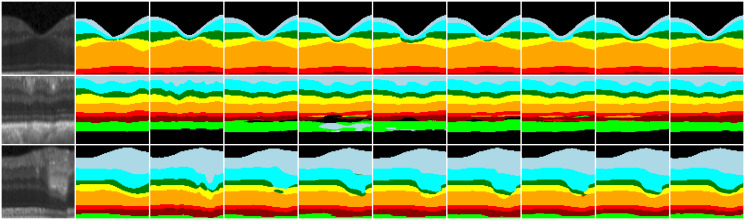
Segmentation results of HCMS dataset. From left to right, the columns display the initial image, ground truth, and the segmentation results of AURA, U-Net, UNet++, Res-UNet, Attention U-Net, ReLayNet, FCRN, and our method, respectively.

**Fig 9:**
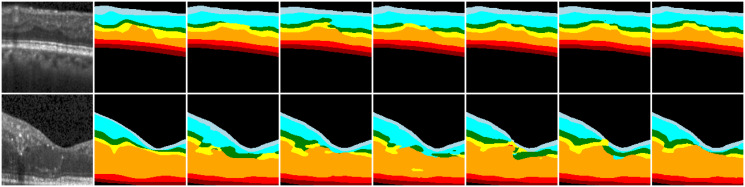
Segmentation results of DUKE dataset. From left to right, the columns display the initial image, ground truth, and the segmentation results of U-Net, UNet++, Res-UNet, Attention U-Net, FCRN, and our method, respectively.

To demonstrate the effectiveness of our method, we provide specific segmentation results on two datasets, as depicted in Figs 8 and 9. The figure showcases various image samples and their corresponding segmentation outputs. The first column represents the original images, while the subsequent columns depict the ground truth and the results obtained from U-Net [7], UNet++ [8], Res-UNet [28], Attention U-Net [17], TransUnet [9], ReLayNet [10], FCRN [19] and our proposed method.

The retinal layer images exhibit numerous depressions and elevated areas, while the OCT images are affected by noise and weak border issues. Fig 8 shows some of the hard-to-split cases for HCMS data. First, the top row displays a depressed region in the retinal layer where the widths of the IPL, INL, and OPL are extremely narrow, leading to disrupted segmentation structures in these areas. The results indicate that AURA effectively preserves the central INL structure, but there may be deviations in the boundary positioning of the concave area, thereby reducing the segmentation accuracy. In contrast, U-Net, Attention U-Net, and RelayNet often fail to capture the central INL. On the other hand, UNet++, Res-UNet, and FCNR can identify these elongated structures, but none of them manage to preserve the original topology effectively. Our method, however, maintains these elongated topologies well. Second, the second line illustrates an uneven, irregular retinal layer with low contrast issues. Traditional methods, when faced with low contrast, struggle to accurately locate boundary points, further resulting in jagged boundaries and a decrease in segmentation accuracy. Mainstream segmentation algorithms struggle to preserve the positional relationships between different retinal layers accurately. Although RelayNet and FCRN, which are designed specifically for retinal segmentation, manage to maintain the approximate hierarchical relationships, there are still mis-segmented pixels. In contrast, our method preserves these details more effectively. Third, the third line illustrates the impact of highlighted regions on the segmentation structure. Traditional methods are affected by high-intensity regions, leading to abnormal protrusions and depressions at the boundaries. The pixel gradients produced by these highlighted areas can lead the network to learn incorrect features. As shown in the figure, the highlighted region disrupts the network’s learning structure for the OPL and ONL layers, whereas only our network successfully maintains the correct structure.

The complex structure of the DUKE dataset we show in Fig 9. In the first line, we highlight a region with weak boundaries. Most mainstream networks struggle to accurately recognize these slender layers, while our method effectively segments the weakly bounded areas. This performance is consistent with the results observed on the HCMS dataset. In the second row, we present images with significant skewing, a result of not flattening the DUKE dataset. The figure reveals a downward prominence in the OPL, which most methods, including ours, struggle to accurately capture. However, the latter three methods, specifically designed for fundus segmentation, perform better than single-task methods. In the depressed region on the right, only our method successfully maintains the hierarchical structure.

### 5.2 Ablation experiments

In our proposed method, we introduce a double task network that aims to achieve target segmentation and boundary regression simultaneously. To effectively leverage the segmentation features and boundary features, we present a spatial attention mechanism that combines feature information from both tasks, enhancing the network’s ability to extract target and boundary features. In the boundary regression branch, we utilize the original labeled TSDF function as the task, which serves as a guide for the network to learn boundary features more effectively. To ensure the network focuses more on the spatial relationships near the boundaries, we set the TSDF threshold to 5. Additionally, adaptive weights are designed to enhance attention to both boundary regions and areas with misclassification. To address the issue of limited training samples in medical imaging, we also propose a channel attention mechanism to prune the model, reducing the risk of network overfitting. This allows for more robust and reliable predictions. In the following sections, we will conduct ablation experiments on each of these improvements to demonstrate their effectiveness.

#### 5.2.1 Ablation experiment for double task

In this Section, we conducted ablation experiments on the improved parts. Firstly, we analyzed the effect of double task networks on model accuracy. Table 3 presents the experimental results of our method compared to Res-UNet, Res-UNet with double head project(DH U-Net), double branch Res-UNet (DB U-Net), double branch Res-UNet with feature concatenation (DB U-Net+Concat) and double branch Res-UNet with SADBIFB(DB U-Net+SADBIFB). It can be observed that the use of double tasks networks in DH U-Net allows the network to capture more boundary features, leading to an improvement in the segmentation accuracy. However, this method does not involve information coupling within the network, which limits the improvement in segmentation accuracy.

**Table 3:** Evaluation of the effectiveness of double task.

Model	RNFL	GCIPL	INL	OPL	ONL	IS	OS-RPE	Dice	IoU
Res-UNet	93.28%	94.92%	88.07%	90.30%	94.96%	86.93%	86.95%	90.86%	93.60%
DH-Res-UNet	93.39%	94.87%	88.02%	90.16%	94.99%	86.73%	**87.26%**	90.89%	93.66%
DB-Res-UNet	93.45%	94.93%	88.04%	90.41%	95.08%	**87.25%**	86.98%	90.93%	93.67%
DB-Res-UNet+Concat	**93.50%**	94.94%	88.15%	**90.48%**	**95.09%**	87.14%	86.80%	90.95%	93.71%
DB-Res-UNet+SADBIFB	93.44%	**94.95%**	**88.24%**	90.40%	94.94%	87.09%	87.18%	**90.99%**	**83.81%**

On the other hand, the DH U-Net+Concat method directly concatenates the features of the segmentation branch and the boundary regression branch, thereby achieving more accurate results and increasing the accuracy.

In this article, we propose the adoption of a feature fusion module based on Spatial Attention to effectively utilize both segmentation features and boundary regression features. By promoting the complementarity of these features, we are able to achieve optimal results with an increased accuracy.

#### 5.2.2 Ablation experiment for boundary regression loss

This section aims to provide a detailed analysis of the objectives of boundary regression. FCRN [19] regresses the boundary coordinates of each column using the cross-entropy loss function. While using only boundaries as regression targets can enhance the network’s ability to extract boundary features, it can also result in significant loss when the predicted results differ from the actual results by just one pixel. This heavy reliance on label results can be problematic for medical imaging, where labels at weak boundaries often contain uncertainty and noise. As a result, the network may overfit to this label noise.

**Fig 10:**
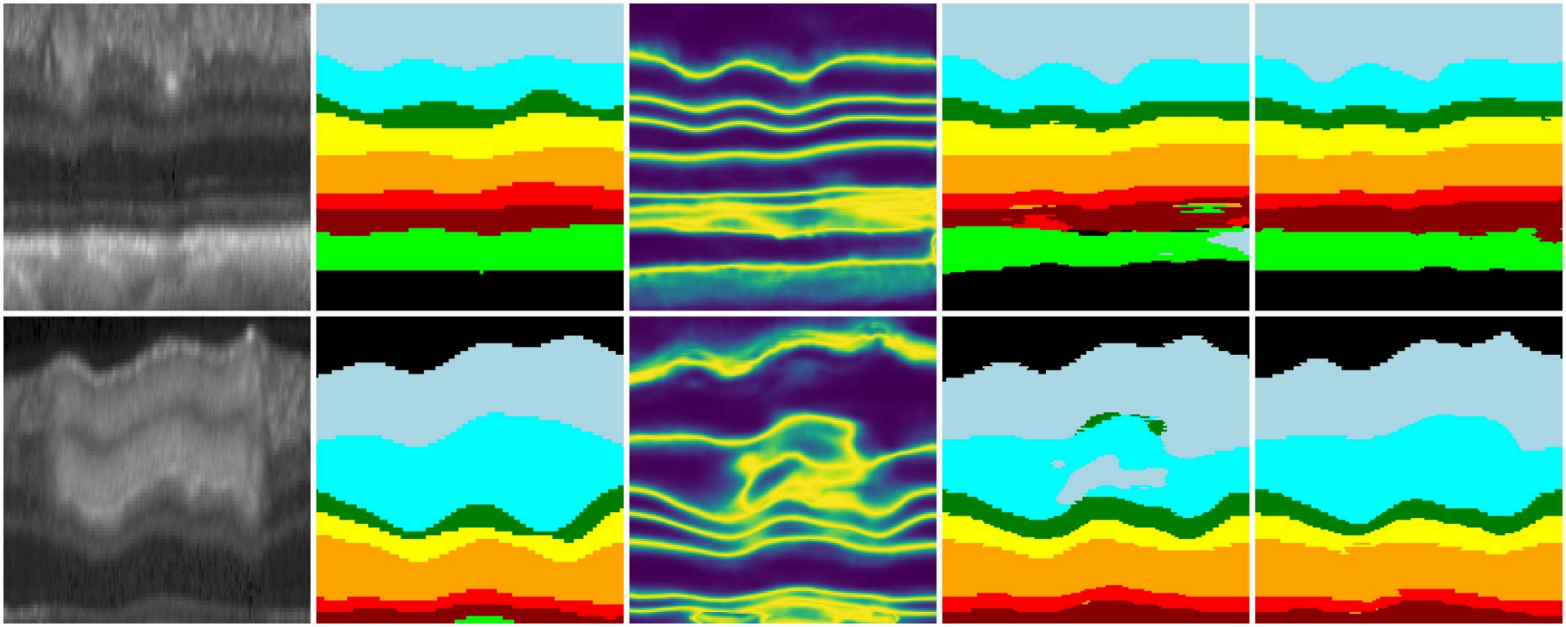
The hotmap of adaptive weight. The figure displays, in order: the original image, labels, adaptive weights, results obtained using TSDF loss, and results obtained using weighted TSDF loss.

To address this issue, Tan et al. [4] propose a regression objective based on soft-argmax on the boundary. This approach effectively reduces the interference of label noise and yields better results. However, due to the isotropy of the convolution kernel, it tends to generate pseudo boundaries at complex boundaries. To overcome this challenge, we introduce a regression objective based on the truncated signed distance function, which effectively minimizes the generation of pseudo boundaries and achieves more accurate results. In our experiments, we observed that the nine TSDF values produced by the boundary regression branch for the same pixel tend to be very similar, particularly near the boundary and in regions where the network has not accurately learned the topology. To address this issue, we designed the adaptive weights described in the main text, which are visualized as shown in Fig 10.

The heat maps corresponding to the adaptive weights, the results obtained using the TSDF loss function, and the results obtained using the weighted TSDF loss function are shown in Fig 10. From the Fig 10, we can observe that the unweighted TSDF loss function fails to capture the correct topology in some weak boundaries and diseased regions. Consequently, the adaptive weights we designed are increased in these areas. Additionally, there is a high response near the boundaries. The weighted TSDF loss function emphasizes these regions, allowing the network to better learn the complex topology.

We performed ablation experiments on several of these loss functions. For the fairness of the experiments, we uniformly use our proposed model for the experiments. Table 4 showcases the final segmentation results obtained using several boundary regression targets. It is evident that our method has improved the IoU by 1.06%.

**Table 4:** Evaluation of the effectiveness of boundary regression target.

Loss	NFL	IPL	INL	OPL	ONL	IS	OS	RPE	Dice	IoU
br_ce_loss	93.03%	94.75%	87.55%	89.86%	94.67%	86.25%	86.01%	91.08%	90.40%	82.90%
soft_argmax_loss	93.37%	94.91%	88.05%	90.42%	95.02%	86.87%	86.87%	91.88%	90.92%	83.27%
tsdf_loss	**93.44%**	94.95%	**88.24%**	**90.40%**	94.94%	**87.09%**	87.18%	91.67%	90.99%	83.81%
weight_tsdf_loss	93.38%	**94.97%**	88.19%	90.36%	**94.95%**	86.84%	**87.83%**	**92.13%**	**91.08%**	**83.96%**

#### 5.2.3 Ablation experiment for pruning

In medical image analysis, the scarcity of calibration data often results in network overfitting and increased computational costs, posing challenges for clinical deployment. Furthermore, the utilization of a dual task model necessitates a larger number of parameters. To address this, RelayNet and FCRN have adopted lightweight configurations, though this approach can reduce the model’s learning capacity. Therefore, network pruning becomes crucial to reduce the number of model parameters and mitigate the risk of overfitting. In this section, we will delve into the performance disparities of networks pre and post pruning, along with comparing the diverse effects resulting from various pruning strategies. Table 5 demonstrates the model accuracy achieved under different pruning strategies. Additionally, we compared the U-Net network with the lightweight networks FCRN, as shown in the table.

**Table 5:** Evaluation of the effectiveness of pruning.

Model	Params	NFL	IPL	INL	OPL	ONL	IS	OS	RPE	Dice	IoU
U-Net	7.85	92.61%	94.19%	86.95%	89.25%	94.32%	85.79%	86.76%	91.51%	90.17%	82.49%
FCRN(tiny)	1.38	93.26%	94.80%	88.07%	90.36%	95.02%	86.78%	87.04%	92.04%	90.92%	83.72%
MTAMN(Ours)	6.64	93.44%	94.95%	88.24%	90.40%	94.94%	87.09%	87.18%	91.67%	90.99%	83.81%
MTAMN+L1	2.12	93.33%	**95.01%**	88.21%	90.46%	94.97%	86.91%	86.76%	91.35%	90.87%	83.61%
MTAMN+L2	2.14	93.41%	95.00%	**88.25%**	90.45%	95.07%	86.93%	87.03%	91.24%	90.92%	83.68%
MTAMNP(Ours)	2.07	**93.45%**	94.92%	88.16%	**90.43%**	**95.10%**	**87.22%**	**87.64%**	**92.27%**	**91.15%**	**84.06%**
	1.39	93.43%	94.97%	88.18%	90.41%	95.08%	86.62%	87.59%	92.23%	91.06%	83.93%

To demonstrate the superiority of the channel attention-based pruning strategy proposed in this article, we conducted a comprehensive comparison with pruning strategies based on L1 and L2 criteria. Our aim was to showcase how our pruning strategy outperforms the others and achieves higher segmentation accuracy. After pruning to achieve a lower parameter count, we observed noteworthy results. Our pruning strategy not only achieved comparable pruning levels but also demonstrated higher segmentation accuracy compared to the other two strategies (L1 and L2). This highlights the effectiveness and efficiency of our proposed channel attention-based pruning strategy.

After model pruning, the segmentation accuracies of various tissues exhibit differential changes, reflecting the tissue-dependent effects of the pruning strategy on model performance. The results show significant improvements in the accuracy of ONL (+0.17%), IS (+0.12%), OS (+0.46%), and RPE (+0.60%), particularly for OS and RPE. This indicates that the pruning strategy effectively reduces redundant parameters and enhances the model’s ability to focus on key feature extraction for these specific tissues. At the same time, NFL (+0.01%) and OPL (+0.03%) demonstrate modest gains, reflecting a degree of robustness and stability, as their segmentation performance remains largely unaffected by pruning.

However, slight declines in the accuracy of IPL (-0.04%) and INL (-0.08%) suggest that pruning may have compromised the model’s ability to capture the finer details of these tissues. These regions likely require richer feature representations, and the reduction in parameters may have led to insufficient feature extraction, impacting segmentation performance.

Overall, the pruning strategy enhances the segmentation of critical tissues while maintaining the model’s overall performance, with notable improvements in key areas such as OS and RPE. This demonstrates that careful parameter pruning can improve segmentation accuracy for specific important tissues while reducing model complexity. However, further optimization of the pruning strategy is necessary to account for the varying sensitivities of different tissues, ensuring a comprehensive improvement in segmentation performance across all tissues.

In our experiments, the choice of pruning ratio *r*% plays a crucial role in model performance. The pruning ratio *r*% determines the percentage of channels removed during the training process, and selecting an appropriate value is essential for balancing the model’s computational efficiency and segmentation accuracy. To ensure the effectiveness of our pruning strategy, we employ a depth-based convolutional kernel grouping approach: the kernels are grouped by depth, and in each group, the bottom *r*% of the least important kernels are pruned. After each pruning step, the remaining model is fine-tuned, and this process is repeated, pruning r% of the kernels in each group for a total of five iterations. The model with the best performance is selected after all rounds of pruning and fine-tuning.

As shown in the experimental results (Table 6), when the pruning ratio is small (e.g., 5%), the model retains a high number of parameters (5.84M) and performs similarly to the baseline across all layers. As the pruning ratio *r*% increases, the model’s parameter count decreases gradually, without a significant drop in performance. For example, when the pruning ratio reaches 20%, the model achieves the best balance, with a Dice score of 91.15% and an IoU of 84.06%, indicating that moderate pruning effectively reduces the number of parameters while maintaining high segmentation accuracy. However, when the pruning ratio is increased further (e.g., 30%), we begin to observe a slight decrease in performance, likely due to the removal of important features in the deeper layers. This suggests that while pruning helps to improve computational efficiency, excessive pruning can lead to the loss of essential information, particularly in deeper feature channels. Based on our experimental results, we conclude that a pruning ratio *r*% between 20% and 25% offers the optimal balance between reducing the number of parameters and maintaining segmentation accuracy. This range allows for significant parameter reduction while preserving key structural and boundary information.

**Table 6:** Evaluation of the effectiveness of the pruning ratio (r%).

Model	r	Params	NFL	IPL	INL	OPL	ONL	IS	OS	RPE	Dice	IoU
MTAMN(Ours)	\	6.64	93.44%	94.95%	88.24%	90.40%	94.94%	87.09%	87.18%	91.67%	90.99%	83.81%
MTAMNP(Ours)	5%	5.84	92.96%	94.63%	87.88%	90.14%	94.89%	86.81%	87.72%	91.90%	90.87%	83.61%
	10%	4.50	93.33%	94.85%	88.04%	90.33%	94.94%	86.57%	87.50%	91.86%	90.93%	83.72%
	15%	3.53	93.25%	94.56%	87.45%	89.93%	94.95%	86.70%	**87.80%**	**92.31%**	90.87%	83.60%
	20%	2.07	**93.45%**	94.92%	88.16%	**90.43%**	**95.10%**	**87.22%**	87.64%	92.27%	**91.15%**	**84.06%**
	25%	1.39	93.43%	**94.97%**	**88.18%**	90.41%	95.08%	86.62%	87.59%	92.23%	91.06%	83.93%
	30%	0.98	93.08%	94.44%	87.78%	90.01%	94.59%	86.31%	86.97%	91.54%	90.59%	83.50%

In conclusion, the results of our experiments clearly indicate that our pruning strategy excels in terms of segmentation accuracy. By considering channel attention, we effectively remove unnecessary channels while maintaining the overall integrity of the segmentation process. We can observe that our parameters are close to FCRN, while IoU has increased by 0.34%.

#### 5.2.4 The summary of ablation experiment

In order to provide a clearer understanding of the improvement in network accuracy achieved through multiple enhancements discussed in this article, we conducted a comprehensive ablation experiment. The results of this experiment are summarized in Table 7.

**Table 7:** The summary of ablation experiment.

Model	SADBIFB	TSDF-Loss	Weight	Prune	Dice	IoU
Res-UNet					90.86%	83.60%
DB-Res-UNet	√				90.93%	83.73%
DB-Res-UNet	√	√			90.99%	83.81%
DB-Res-UNet	√	√	√		91.08%	83.96%
DB-Res-UNet	√	√	√	√	**91.15%**	**84.06%**

From the table, it is evident that our dual task network incorporates boundary regression tasks based on TSDF, enhancing the network’s ability to acquire boundary features. Additionally, we employ a coupling module that utilizes spatial attention to facilitate feature fusion between the two tasks, enabling target segmentation and boundary regression to complement each other. Furthermore, our pruning strategy, which is based on channel attention, not only reduces the number of model parameters but also mitigates the risk of network overfitting.

The experimental results demonstrate that our method improves the average segmentation accuracy in Dice by 0.29% and IoU by 0.47%, compared to the Res-UNet network.

## 6 Conclusion and discussion

This article presents a new dual branch network designed to improve the accuracy of Retinal OCT Segmentation. The network achieves target segmentation and boundary regression simultaneously, utilizing a truncated signed distance function to reduce the impact of weak boundaries. Meanwhile, an adaptive weight was designed based on TSDF. Additionally, it introduces a spatial attention coupling module to integrate segmentation and boundary regression features, enhancing their complementarity. Furthermore, the article proposes a pruning module based on attention mechanism to reduce model parameters and the risk of overfitting.

We believe that this article primarily focuses on learning TSDF as a network learning task. In the future, we aim to explore how to better integrate level set algorithms with deep learning, leveraging the bridge established in this work. Additionally, Due to a lack of labeled medical image data, one of our future research focuses is on developing a semi-supervised model to leverage unlabeled data for improved network feature extraction of targets.

Our proposed model enables more accurate OCT segmentation results, providing quantitative support for subsequent disease diagnosis (e.g., tissue thickness measurements). However, in actual clinical practice, certain pathologies such as macular edema, glaucoma, and optic nerve diseases can lead to abnormal tissue thickness. Significant thickness abnormalities can be easily detected based on the segmentation results. However, subtle thickness changes are also associated with certain diseases. In future research, we will further explore the relationship between minor thickness variations and disease, providing more precise diagnostic references for physicians to comprehensively assess the condition and develop more effective treatment plans.

## Supporting information

S1 File (DOCX)

S2 File (PDF)
