# Supplementary material for: A weak edge estimation based multi-task neural network for OCT segmentation
Source: PLoS One. 2025 Jan 3;20(1):e0316089. doi: 10.1371/journal.pone.0316089 (PMC11698417; doi:10.1371/journal.pone.0316089)
Supplement: S1 File — (DOCX) [file pone.0316089.s001.docx]

**Supporting Information**

The data utilized in this study is sourced from two publicly available datasets. The first dataset is the HCMS dataset, which can be accessed at https://iacl.ece.jhu.edu/index.php?title=Resources. The second dataset is the DUKE dataset, available at https://people.duke.edu/~sf59/Chiu_BOE_2014_dataset.htm. Both datasets provide critical data for the analyses conducted in this research and support the study’s conclusions.

Table1: Results of Five-Fold Cross-Validation on the HCMS Dataset Using MTAMN(Ours)

| Iteration | NFL | IPL | INL | OPL | ONL | IS | OS | RPE | Dice | IoU |
| --- | --- | --- | --- | --- | --- | --- | --- | --- | --- | --- |
| KF_1 | 93.52% | 94.90% | 88.02% | 90.32% | 95.02% | 87.07% | 87.66% | 91.56% | 91.01% | 83.84% |
| KF_2 | 93.54% | 94.92% | 88.07% | 90.33% | 95.08% | 86.91% | 87.20% | 91.82% | 90.98% | 83.80% |
| KF_3 | 93.52% | 94.94% | 87.95% | 90.21% | 95.10% | 87.29% | 87.43% | 91.71% | 91.02% | 83.86% |
| KF_4 | 93.35% | 94.91% | 88.05% | 90.40% | 95.09% | 87.07% | 87.65% | 92.31% | 91.10% | 84.01% |
| KF_5 | 93.25% | 95.10% | 89.10% | 90.75% | 94.39% | 87.11% | 85.94% | 90.93% | 90.82% | 83.54% |
| AVG | 93.44% | 94.95% | 88.24% | 90.40% | 94.94% | 87.09% | 87.18% | 91.67% | 90.99% | 83.81% |

Table2: Results of Five-Fold Cross-Validation on the HCMS Dataset Using MTAMNP(Ours)

| Iteration | NFL | IPL | INL | OPL | ONL | IS | OS | RPE | Dice | IoU |
| --- | --- | --- | --- | --- | --- | --- | --- | --- | --- | --- |
| KF_1 | 93.63% | 95.09% | 88.17% | 90.51% | 95.21% | 87.22% | 87.85% | 91.74% | 91.18% | 84.09% |
| KF_2 | 93.66% | 95.10% | 88.19% | 90.45% | 95.20% | 87.10% | 87.38% | 91.97% | 91.13% | 84.01% |
| KF_3 | 93.67% | 95.11% | 88.09% | 90.35% | 95.26% | 87.41% | 87.55% | 91.88% | 91.17% | 84.07% |
| KF_4 | 93.45% | 95.08% | 88.18% | 90.53% | 95.26% | 87.20% | 87.78% | 92.43% | 91.24% | 84.19% |
| KF_5 | 92.85% | 94.20% | 88.18% | 90.31% | 94.58% | 87.15% | 87.62% | 93.32% | 91.03% | 83.96% |
| AVG | 93.45% | 94.92% | 88.16% | 90.43% | 95.10% | 87.22% | 87.64% | 92.27% | 91.15% | 84.06% |

Table3: Results of Five-Fold Cross-Validation on the DUKE Dataset Using MTAMNP(Ours)

| Iteration | RNFL | GCIPL | INL | OPL | ONL | IS | OS-RPE | Dice | IoU |
| --- | --- | --- | --- | --- | --- | --- | --- | --- | --- |
| KF_1 | 92.64% | 95.92% | 90.45% | 87.81% | 97.33% | 93.06% | 95.67% | 93.27% | 87.17% |
| KF_2 | 92.60% | 95.83% | 90.30% | 87.65% | 97.33% | 93.12% | 95.71% | 93.22% | 87.12% |
| KF_3 | 91.87% | 95.16% | 89.66% | 86.96% | 96.55% | 92.40% | 94.97% | 92.51% | 86.38% |
| KF_4 | 91.07% | 94.36% | 88.84% | 86.16% | 95.72% | 91.59% | 94.20% | 91.71% | 85.57% |
| KF_5 | 92.09% | 95.04% | 89.64% | 87.03% | 96.45% | 92.49% | 95.14% | 92.55% | 86.45% |
| AVG | 92.05% | 95.26% | 89.78% | 87.12% | 96.68% | 92.53% | 95.14% | 92.65% | 86.54% |

I have uploaded all relevant images and data to the following public repository: https://figshare.com/articles/dataset/__OCT__/27369336.
