# Supplementary material for: A weak edge estimation based multi-task neural network for OCT segmentation
Source: PLoS One. 2025 Jan 3;20(1):e0316089. doi: 10.1371/journal.pone.0316089 (PMC11698417; doi:10.1371/journal.pone.0316089)
Supplement: S2 File — (PDF) [file pone.0316089.s002.pdf]

Request for Authorship Change(s)

Authorship change requests are subject to PLOS approval. All applicable sections of this form must be completed in full before we will review your request. Please note that per the PLOS Authorship Policy we require agreement from all authors (including those being added or removed) for any changes to a submission's author list.

PLOS does not generally consider requests to add or remove authors after editorial acceptance; any late-stage requests require approval by the journal's editorial team.

In some cases, depending on the nature, timing, and/or extent of authorship changes requested, PLOS may require verification of the updated author list and contributions by an official at the authors' institution(s). Difficulties reaching an institutional official may delay your submission's peer review or publication.

Section 1. Confirm adherence to PLOS policies

☑ Check here to confirm you have read the *PLOS* Authorship policy, and that the updated author list requested in this form complies in full with the criteria outlined in the Authorship Requirements section of our policy. Key points from the policy that pertain to authorship criteria are summarized here:

Everyone listed as an author must meet all criteria for authorship, and everyone who meets the criteria for authorship should be listed as an author.

Authorship criteria for *PLOS Medicine*:

1. Conception and design of the work, acquisition of data, or analysis and interpretation of data; and
2. Drafting the article or revising it critically for important intellectual content; and
3. Final approval of the version to be published; and
4. Agreement to be accountable for all aspects of the work

Authorship criteria for all other PLOS journals:

1. Substantial contributions to one or more of the following: conception or design of the work; acquisition, analysis, or interpretation of data; creation of new software used in the work; drafting or substantially revising the article; and
2. Approved the submitted version (and any substantially modified version that involves the author's contribution to the study); and
3. Agrees to be personally accountable for the author's own contributions AND to ensure that questions related to the accuracy or integrity of any part of the work are appropriately investigated, resolved, and the resolution documented in the literature.

Any contributions that do not meet authorship criteria should be discussed in the Acknowledgments section of the manuscript; authors are responsible for obtaining permission to name individuals in the Acknowledgments.

Payment of Article Processing Charges (APC) does not qualify for authorship.

☑ Check here to confirm that all authors (including those to be added or removed) consent to the requested changes.

☑ Check here to confirm that you have read PLOS policies on Competing Interests and Funding Disclosures. Complete the following table to describe any updates needed to your article's disclosures.

	Updates needed to the submission's disclosure statement	No change needed
**Competing Interests**		☑
**Funding**		☑

Section 2. Final manuscript information

**Manuscript number**<br>e.g., PONE-D-17-00000	PONE-D-24-37111R4
**Complete author list, in correct order**<br>Please note any equal contributors with asterisks (*) or hashes (#)	Fan Yang, Pu Chen, Shiqi Lin, Tianming Zhan, Xunning Hong, Yunjie Chen
**Financial Disclosure** – including any additions/deletions necessary due to the change in authorship	
**Competing Interests** – including any additions/deletions necessary due to the change in authorship	
**Acknowledgments statement**<br>Please acknowledge any removed authors if they contributed to the study in any way, as well as members of any author groups who do not meet our authorship criteria.	

Section 3. Requests to add authors

Individual author addition #1

**Full name**	Xunning Hong
**Email address**	hongxunning@sina.com
**Full affiliation**	The First Affiliated Hospital of Nanjing Medical University, Nanjing, Jiangsu, China
**Briefly describe the reason for adding this author, and explain why they were not included on the submission previously.**	We would like to request the inclusion of Xunning Hong as a co-author due to his key role in the validation of our model. The validation is being conducted at Jiangsu Provincial People's Hospital (also known as The First Affiliated Hospital of Nanjing Medical University), where Xunning Hong has assisted and overseen the process. At the time of the initial submission, the validation work was still ongoing, and Xunning Hong's contributions had not yet reached the level required for authorship. However, with the progress of the validation, his substantial involvement now warrants his inclusion as a co-author.

Confirm that this person contributed to **all** of the authorship criteria for the relevant PLOS journal (see above).	☑
**Tick all relevant boxes to indicate this individual's specific contributions:**	
Conceptualization	☐
Data Curation	☐
Formal Analysis	☐
Funding Acquisition	☐
Investigation	☐
Methodology	☐
Project Administration	☐
Resources	☐
Software	☐
Supervision	☐
Validation	☑
Visualization	☐
Writing – Original Draft Preparation	☐
Writing – Review & Editing	☐

Individual author addition #2 (if applicable)

**Full name**	
**Email address**	
**Full affiliation**	
**Briefly describe the reason for adding this author, and explain why they were not included on the submission previously.**	

Confirm that this person contributed to **all** of the authorship criteria for the relevant PLOS journal (see above).	☐
**Specific contributions:**	
Conceptualization	☐
Data Curation	☐
Formal Analysis	☐
Funding Acquisition	☐
Investigation	☐
Methodology	☐
Project Administration	☐
Resources	☐
Software	☐
Supervision	☐
Validation	☐
Visualization	☐
Writing – Original Draft Preparation	☐
Writing – Review & Editing	☐

Author group addition (if applicable)

**Group or consortium name**	
**Author who represents group**	
**Briefly describe the reason for adding this author group, and explain why they were not included on the submission previously.**	

Section 4. Requests to remove authors

Author removal #1

**Full name**	
**Email address**	
**Briefly describe the reason for removing this author at this time given their stated contributions to the manuscript and/or research.**	

**Which authorship criteria have NOT been met by this individual?** (Refer to the criteria numbers in Section 1 of this form)	
Are you updating the article's Acknowledgments to credit this person's contributions?	Yes
If yes, did they agree to be listed in the Acknowledgments?	Yes

If no, explain why you no longer wish to credit this individual for the work attributed to them in the Contributions listings provided previously.

Author removal #2 (if applicable)

**Full name**	
**Email address**	
**Briefly describe the reason for removing this author at this time given their stated contributions to the manuscript and/or research.**	

**Which authorship criteria have NOT been met by this individual?** (Refer to the criteria numbers in Section 1 of this form).	
Are you updating the article's Acknowledgments to credit this person's contributions?	Yes
If yes, did they agree to be listed in the Acknowledgments?	Yes
If no, explain why you no longer wish to credit this individual for the work attributed to them in the Contributions listings provided previously.	

Section 5. Requests for changes to author order

**Prior author list**	
**Requested author list**	
**Briefly describe the reason for requesting a change in author order at this time.**	

**If any authors' contributions need to be updated, provide the affected authors' names and their updated contributions listings here.** Contributions should be listed using the CRediT Taxonomy terms listed below; list all terms that apply.<br><br>If no changes are needed, enter "N/A" in the response box.	

CRediT Taxonomy for author contributions

- Conceptualization
- Data Curation
- Formal Analysis
- Funding Acquisition
- Investigation
- Methodology
- Project Administration
- Resources
- Software
- Supervision
- Validation
- Visualization
- Writing – Original Draft Preparation
- Writing – Review & Editing
